# Does changing from a teacher-centered to a learner-centered context promote self-regulated learning: a qualitative study in a Japanese undergraduate setting

**DOI:** 10.1186/s12909-019-1550-x

**Published:** 2019-05-17

**Authors:** Yasushi Matsuyama, Motoyuki Nakaya, Hitoaki Okazaki, Adam Jon Lebowitz, Jimmie Leppink, Cees van der Vleuten

**Affiliations:** 1 Medical Education Center, Jichi Medical University, 3311-1 Yakushiji, Shimotsuke, Tochigi, 329-0498 Japan; 2 The department of Psychology and Human Developmental sciences, Nagoya University, Furo-cho, Chikusa-ku, Nagoya, Japan; 3 Department of General Education, Jichi Medical University, 3311-1 Yakushiji, Shimotsuke, Tochigi, Japan; 4 Department of Educational Development and Research, Faculty of Health, Medicine, and Life Sciences, Maastricht University, 6200 MD Maastricht, The Netherlands

**Keywords:** Self-regulated learning, Teacher-centered learning, Learner-centered learning, Curriculum reform, Undergraduate education

## Abstract

**Background:**

Previous studies indicate that a teacher-centered context could hinder undergraduates from self-regulated learning (SRL), whereas a learner-centered context could promote SRL. However, SRL development between a teacher-centered and a learner-centered context has not directly compared in undergraduate settings. Also, it is still unclear how a contextual change toward learner-centered learning could influence SRL in students, who are strongly accustomed to teacher-centered learning.

**Methods:**

We conducted three focus groups that examined 13 Japanese medical students who left a traditional curriculum composed of didactic lectures and frequent summative tests and entered a seven-month elective course (Free Course Student Doctor or FCSD). The FCSD emphasizes student-designed individualized learning with support and formative feedback from mentors chosen by students’ preference. We also conducted two focus groups that examined 7 students who remained in the teacher-centered curriculum during the same period. Students were asked to discuss their 1) motivation, 2) learning strategies, and 3) self-reflection on self-study before and during the period. Data were analyzed using thematic analysis and code comparison between the two cohorts.

**Results:**

The non-FCSD participants described their motivational status as being one among a crowd set by the teacher’s yardstick. Their reflection focused on minimizing the gap between themselves and the teacher-set yardstick with strategies considered monotonous and homogeneous (e.g. memorization). FCSD participants described losing the teacher-set yardstick and constructing their future self-image as an alternative yardstick. They compared gaps between their present status and future self-image by self-reflection. To fill these gaps, they actively employed learning strategies used by doctors or mentors, leading to diversification of their learning strategies.

**Conclusions:**

A contextual change toward learner-centered learning could promote SRL even in students strongly accustomed to teacher-centered learning. In the learner-centered context, students began to construct their self-image, conduct self-reflection, and seek diverse learning strategies by referring to future ‘self’ models.

**Electronic supplementary material:**

The online version of this article (10.1186/s12909-019-1550-x) contains supplementary material, which is available to authorized users.

## Background

Because clinical knowledge is rapidly advancing, doctors are expected to self-regulate their learning and update their knowledge autonomously in less structured learning settings in medical practice [1–4].

Self-regulated learning (SRL) is defined as learners’ active participation in their own learning process from metacognitive, motivational, and behavioral perspectives [5]. SRL has been theorized as an orderly, cyclical (meta) cognitive process. For instance, Zimmerman described SRL as a cyclical process composed of three phases. In the forethought phase, learners set learning goals and choose a strategy for attaining goals. In the performance phase, learners monitor and control their behavior to attain goals. In the self-reflection phase, learners formulate new learning goals and strategies for similar situations in future [6, 7].

Now, SRL is considered a key competence for medical students, because residency training cannot prepare residents for every challenge their qualification brings [8]. Furthermore, SRL obtained during undergraduate education could lead to life-long learning [4]. Therefore, lack of readiness to engage in SRL resulting from the undergraduate education system is problematic.

### Contextual factors influencing SRL

Several recent studies emphasize learning context determines whether learners engage in SRL. Brydges & Butler [3] summarized contextual factors influencing SRL: At the broadest level, from learning expectations from cultural and social communities; within learning environments, from pedagogical approaches, specific activities and tasks assigned, learning support, and types of feedback or evaluation. Van Houten-Schat et al. [9] specifically shed light on contextual factors influencing SRL in the clinical environment, such as available time, characteristics of learning environment (work climate, engagement in team), and patient-related factors.

In a study comparing SRL in self-study between undergraduates in a teacher-centered curriculum and physicians in a rural clinical setting, Matsuyama et al. [10] identified contextual factors that may hinder SRL in a teacher-centered curriculum. They included students’ preference to stay close to fellow students, and engaging in monotonous and homogeneous strategies to avoid failing teachers’ assessments. However, postgraduate rural contexts did not keep those learners from being self-regulated. They achieved self-regulation in self-study via 1) awareness of their own unique identity in the learning community, 2) high-stakes tasks which require full responsibility for learning, and 3) experience of coping strategies to complete these high-stakes tasks. Another article reports possible negative effects of teacher-centered undergraduate curriculum on SRL [11]. This demonstrates decrease in cognitive strategic use and self-regulation and increase in anxiety over teacher-centered lectures and summative tests over time.

Moreover, one recent article reveals possible effects of a shift toward a learner-centered context on SRL. It shows the introduction of individualized learning plans with support of mentors during four-week clinical clerkship improved self-efficacy and self-regulation among undergraduates [12]. Taking these results into consideration, to foster SRL in undergraduates in preparation for postgraduate training, a learner-centered context might be more beneficial than teacher-centered. However, there is no investigation directly comparing effects on SRL between a learner-centered context and a teacher-centered context in undergraduate settings.

### Challenges when changing to a learner-centered context in a teacher-centered culture

Medical curriculum reforms from teacher-centered learning to learner-centered learning are proceeding worldwide, based on evidence and theories established mainly in the Western world [13]. In the midst of reformation, contextual changes from teacher-centered to learner-centered learning could challenge students, who are strongly accustomed to teacher-centered education culture [14–17].

For example, teacher-centered education culture is still reported in East Asia or “China and the countries that were heavily influenced by its culture, most notably Japan and Korea” [18]. Traditionally, East Asian education culture is often referred to as Confucian-heritage education where virtue is achieved primarily by learning from teachers and imitating their attitudes [19, 20]. Even today, there is still a notable emphasis in primary and secondary East Asian education on reproducing teachers and textbook information. Moreover, in pre-university education, students are urged to attain higher grade point averages and rankings to enable them to attend prestigious universities for future success [19]. Overall, entrance examinations for universities emphasize accuracy in the reproduction of informational content. Tutors in preparatory cram schools devise strategies to repetitively review past lessons (such as past examination papers) to prepare for entrance examinations [21]. This pedagogy may cause East Asian medical students to be fully accustomed to teacher-centered education when entering universities.

This entails challenges when these medical students encounter curriculum reforms from a teacher-centered to a learner-centered context. Yoshioka et al. [14] report that Japanese medical students have difficulty extracting problems without instruction from teachers in problem-based learning (PBL) in a learner-centered context. Frambach et al. [15] report that medical students in Hong Kong had anxiety about PBL discussions and asked for explanatory lectures from teachers.

As the introduction of learner-centered philosophy challenges learners in teacher-centered cultures in various parts of the world [16, 17], educators can explore how a contextual change toward learner-centered learning could influence SRL in students, who are strongly accustomed to teacher-centered learning, as a general issue.

### Present study

The aim of this study was to explore whether contextual changes from a teacher-centered to a learner-centered learning could improve SRL in an undergraduate setting. To clarify the aim of this study, we formulated two related research questions: 1) Does change from a teacher-centered to a learner-centered context stimulate SRL; and 2) how does SRL develop during transition from a teacher-centered to a learner-centered context. To address these research questions, we compared self-regulation in learning between medical students who were experiencing contextual change from teacher-centered to learner-centered learning and those who remained in teacher-centered curriculum at the same school year period.

The study was approved by Jichi Medical University Clinical Research Ethics Committee (reference number: 15–154). Informed consent was obtained from all participants. Data collection was conducted from July 2017 to January 2018. Data analysis was conducted in parallel with data collection from November 2017 to March 2018.

## Methods

### Settings

#### Current Jichi Medical University curriculum as teacher-centered learning context

Jichi Medical University (JMU) in Japan is a publicly funded medical school whose mission is to increase the number of rural doctors and employ them nationwide. In the current curriculum at JMU (Table 1), students finish lectures on almost every subject in basic and clinical medicine before end of Year 3. From Year 4 to May in Year 6, students are permitted to participate in a clinical clerkship, during which they receive training centered mainly on taking patient histories and providing physical examination, but teachers prefer to provide relevant information via lectures rather than the medical practice. Even though they are in the clinical clerkship, they are mainly assessed by an annual comprehensive summative test (Year 4 and 5 *Sougouhantei-Shiken*), which require them to recall knowledge conveyed by teachers. Moreover, Year 6 students must receive didactic lectures on 17 clinical subjects, and take and pass summative tests for each clinical subject. JMU has a good reputation for its high pass rate in exams [22]. However, a previous study [10] revealed that medical students at JMU perceived the current curriculum as teacher-centered and test-oriented, and teacher judgements based on their test performance neglected their individual learning processes.

**Table 1:**
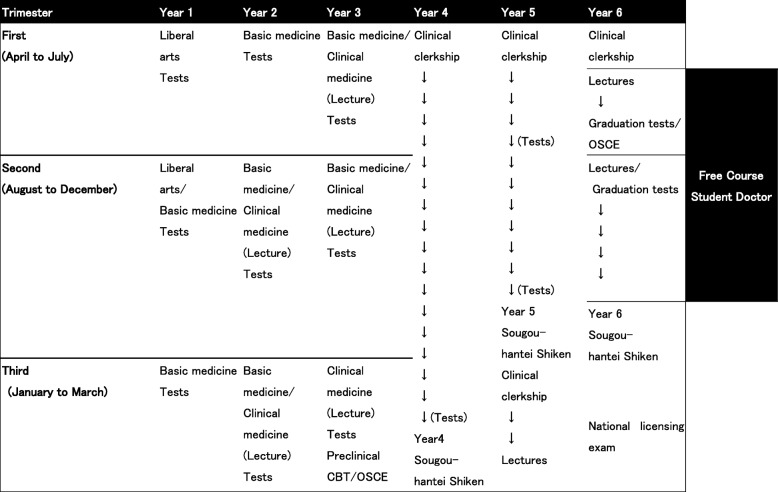
Undergraduate curriculum at Jichi Medical University and the Free Course Student Doctor period

Note. CBT - Computer-based test; OSCE - Objective Structured Clinical Examination

#### A novel student-designed elective course as learner-centered context

In 2011, the Education Board at JMU designed a novel program named the Free Course Student Doctor (FCSD). Students, whose scores on the annual summative test at Year 5 are higher than the average scores of Year 6 students, are considered knowledgeable enough to pass all summative tests in Year 6 and the national licensing exam to qualify them as doctors. For these advanced students, the Board has established an elective course with individualized learning plans with support and formative feedback from mentors. For 7 months, the FCSD allows students to study any subject they like at any institution throughout the world and are exempt from all didactic lectures and summative tests for 17 clinical subjects (Table 1). With the help of mentors who were also chosen in accordance with student requests, students can individually design seven-month plans pertaining what subjects, which institutions, when, and how long to study. FCSD participants (FCSDs) are asked to report their learning activities via e-mails on a weekly basis and they receive formative feedback mainly from mentors. Of the 79 students eligible since its implementation, 59 participated in the FCSD while 20 chose to continue the conventional Year 6 curriculum. Of these 59, we examined the learning experiences of 39 students who participated in the FCSD from 2011 to 2015 [23]. Results showed that the FCSDs successfully selected subjects that they wanted to study and found institutions based on their preference. All participants selected work-based learning in a variety of departments and institutions. They received feedback individually from corresponding doctors in the medical practice and also formative feedback in weekly reports from their mentors. All but one selected Japanese institutions not only in medical universities but also in hospitals and clinics, while 30 of 39 FCSDs studied in non-East Asian countries (mainly Western countries). As a rule, the FCSD students were fully exempt from lecture-based education or summative graduation tests. The schedule of one FCSD participant is noted as an example in Table 2.

**Table 2:** The Free Course Student Doctor course: Each student can choose learning subjects, institutions and learning contents by themselves with mentor assistance. They experience work-based learning and receive formative feedback

Student: A 24-year-old male in the 2014 cohort Mentor: A chief professor at the department of general internal medicine in Jichi Medical University			
Date	Learning subjects	Institutions	Main learning contents
May	Emergency medicine	A public emergency medical center in the student’s home prefecture in Japan	The first aid for various emergent diseases
June	(1) Infectious diseases (2) General internal medicine	(1) Jichi Medical University (2) A private rural hospital in Japan	(1) In-patient management (2) Management for common diseases
July	Clinical anatomy	Jichi Medical University	Anatomy practice
August	Intensive care	A public medical university in Japan	Advanced intensive care
September	(1) General internal medicine (2) Ultrasonography	(1) A private hospital in Tokyo (2) Jichi Medical University	(1) Management for common diseases (2) Practical skills for ultrasound examinations
October	General internal medicine	Jichi Medical University	Management for complicated cases
November	Family medicine	The department of family medicine in a medical university in the US	Total health care, the role of family physicians in the US

These results indicate that the learning context of FCSD is far removed from the conventional teacher-centered curriculum prevalent in East Asian medical universities. Moreover, we believe the FCSD context corresponds to principles of learner-centered learning proposed by Brandes & Ginnes [24]. First, decision making in all their learning plans entails *learners’ active participation* and *high responsibility in their own learning*. Second, student-designed plans and formative feedback involving preferable tutors are useful platforms to promote *teacher’s role as facilitator*. Third, full exemption from lectures, written tests, and various opportunities in work-based learning ensures *learners’ integrative experience* that stimulates not only cognitive but also affective domains.

### Participants and design

In this study, we focused on FCSDs to explore change in self-regulation on self-study in 7 months of the learner-centered context. We also enrolled students eligible for the FCSD program but elected to remain in the conventional teacher-centered Year 6 curriculum (non-FCSDs), because we believed the comparison between these two cohorts was needed to certify the effects of the FCSD context on SRL.

We employed FCSDs and non-FCSDs between 2015 and 2017 who were in Year 6, post-graduate year (PGY) 1 and 2 during the research period for this study. We excluded participants from before 2015 because recalling learning experience from over 2 years ago was considered problematic.

We invited them to participate in a focus group via e-mail, and all those who agreed were enrolled. We continuously asked all candidates (30 FCSDs and 10 non-FCSDs) to participate until we received agreement or denial of enrollment from them. Eventually, in the first iteration of the focus groups, five FCSDs (four PGY 2 doctors and one PGY 1 doctor), and four non-FCSDs (four PGY 2 doctors) were enrolled. In the second iteration, four FCSDs (4 Year 6 students) and three non-FCSDs (2 Year 6 students and one PGY 2 doctor) were enrolled. In the third iteration, four FCSDs (4 Year 6 students) were enrolled.

### Materials

We collected qualitative data due to the following reasons. First, the study was conducted in a teacher-centered East Asian culture [14, 15, 19], so it was difficult to employ a sufficient number of students, who were surely in learner-centered contexts other than the FCSD, for quantitative study. Second, qualitative research is best suited to developing a detailed understanding of a central phenomenon of study difficult to transform into variables [25]. Therefore, we believed that a qualitative approach could more vividly clarify the contrast in learners’ SRL between those with a contextual change from a teacher-centered to a learner-centered learning and those staying at the teacher-centered curriculum than a quantitative approach.

In recent years, several scholars have emphasized the significance of objective and real-time process-oriented assessment methods such as microanalysis rather than self-recollection or self-assessment procedures to explore SRL [26, 27]. However, we thought real-time assessments might interfere with self-study because participants, especially in teacher-centered culture, might feel pressure or even pretend to do well during assessment. We intended to keep the FCSD context away from assessment-dominated cultures, therefore, used data collection methods in a retrospective manner.

### Procedures

Focus groups maximize the enrichment of expression and exchange of information on mutual topics, particularly when degree of familiarity with the topic is uniform and power relations between the participants are weak [28]. Therefore, we found focus groups suitable to acquiring qualitative data from groups in which students underwent the same learning activity (self-study of clinical knowledge) in the same setting (contextual transition or continuance).

The FCSDs and non-FCSDs were separately invited to participate in focus groups. Focus groups using PGY 1 and 2 doctors were conducted over Skype®, because the participants were busy in their residency programs in different institutions throughout Japan and had difficulty in scheduling face-to-face meetings. Focus groups using only Year 6 medical students took place in a face-to-face manner at JMU. Compared with face-to-face meetings, the internet connection during Skype® meetings might influence the frequency of participants statements or verbatim accuracy. However, there was no serious connection problem nor discrepancy between recorded and given statements during Skype® meetings.

After informed consent was obtained, a 90–120-min focus group was conducted. All conversations during the session were recorded and transcribed by research assistants. Participants were not identified in order to guarantee anonymity. The focus group was implemented using three questions prepared beforehand.

- *Q1. Could you recollect your experience of self-study (of medical knowledge) during the FCSD or the same period in the conventional curriculum?*
- *Q2. During that period, how did you motivate yourself, what strategies did you apply to learning, and how did you assess your understanding?*
- *Q3. Between before and during the period, did you experience any change in terms of how you motivated yourself and the strategies you applied to learning and to assessing your understanding?*

Among the three questions, the third question for FCSDs was considered to be most important to explore changes in SRL when the same learners experience shift from a teacher-centered to a learner-centered context. Rather, purpose of the first and the second question was to prompt FCSDs and non-FCSDs to recall their self-study experience, and articulate three aspects of SRL. These were self-motivation, learning strategies, and metacognition [5] during the FCSD course, or the same period of didactic lectures and summative tests, respectively.

In focus groups, we have specifically inquired self-study for medical knowledge as a learning activity for the following reasons. First, knowledge acquisition is a common task for students of the two groups compared in this study. Second, our previous study [10] used the similar learning content and successfully illuminated the differences in SRL between the teacher-centered curriculum and the postgraduate rural setting.

### Analysis

From a constructivist paradigm in which ‘reality’ is subjective and context-specific, and multiple truths are constructed by and between people [29], we employed constructivist thematic analysis, which examines ‘the ways in which events, realities, meanings, experiences and so on are the effects of a range of discourses operating within society’ [30]. We viewed this method as suitable for analysis of data from the focus groups, where discourse takes place among participants in the same learning context.

We inductively coded anonymized transcripts of the Japanese scripts from the two groups. Initial coding was conducted by the two lead Japanese researchers, a medical educator (YM) and an education psychologist (MN). Both were experienced in the conduct of qualitative studies relevant to SRL. The analysis was conducted in accordance with the six phases of Braun and Clarke’s thematic analysis [30]. The transcripts were thoroughly read and analyzed using an inductive coding approach until agreement on coding was achieved through Skype® meetings between the pair.

In the coding process, we utilized terms described in the Motivated Strategies for Learning Questionnaire (MSLQ) [31]. MSLQ is composed of 81 items which quantify the scales of nine types of SRL strategies (rehearsal, elaboration, organization, critical thinking, metacognitive self-regulation, time and study environment, effort regulation, peer learning, and help seeking), and six variables of motivation states (intrinsic goal orientation, extrinsic goal orientation, task value, control of learning beliefs, self-efficacy for learning and performance, and test anxiety).

In initial coding, we firstly coded participants’ transcripts for Q1 and Q2 in each group by focusing on how self-motivation, behaviors and reflection took place during the FCSD and the conventional curriculum. Second, we coded their verbatim for Q3 in each group by focusing on how participants in each focus group perceived the changes in self-motivation, behaviors, and reflection before and during the 7 months.

After coding agreement, codes and representative statements were translated into English by an American professor living in Japan, who speaks both English and Japanese (AJL). In the final phase, the other authors (HO in Japan and JL and CV in the Netherlands) joined the discussion. We compared codes between students who experienced the shift from teacher-centered to FCSD context and those in the same school year who continuously remained in the teacher-centered curriculum, and a higher-level synthesis of the codes eventually resulted in major themes.

## Results

The result section is structured according to the research questions. Findings are noted with representative statements from focus groups and their reference numbers (e.g. P3–77). Representative codes and statements written in Japanese and English are included within Additional file 1.

### Does change from a teacher-centered to a learner-centered context stimulate SRL?

To address the first research question, we focused on FCSDs’ perceptions toward changes in self-motivation, behaviors, and reflection between before and during the 7 months. These were mainly articulated in focus groups for Q3, or as the question: ‘Between *before* and *during* the period, did you experience any change in terms of how you motivated yourself, and the strategies you applied to learning and to assessing your understanding?’

While recalling the 7 months during the FCSD, FCSDs looked back on their previous selves before entering the FCSD. They perceived themselves as part of a group of elite test takers, who were preprocessed with the teacher’s assessment standard, or yardstick. Then, they described contextual changes experienced in the FCSD as *liberation or no yardstick*, which resulted in confusion.

*‘There's no yardstick to measure your outcomes. We're all part of that group of elite test takers, so at the beginning when you're liberated from that framework, it's really mind-boggling, confusing.' (FCSD, P3–77)*

However, the FCSDs strived to find an alternative indicator by measuring distance between their current ability and their future self-image. To measure this distance, on one hand they actively employed self-reflection to recognize their current status, and on the other autonomously created their achievable self-image. The FCSDs searched for hints that would help them realize their achievable self-image by employing careful and attentive observation of model doctors and an active approach to communicating with mentors in weekly formative feedback (help seeking in MSLQ).

*'I thought I'd find a doctor who could be a model for me, who knew how to write really good patient reports and was really good with them on a one-on-one basis, because I knew there just had to be one like that.' (FCSD, P1–65)*

At the same time, they focused on learning strategies used by model professionals and attempted to adapt them to their own self-study.

*‘I could also see the profs screwing-up sometimes and getting anxious about their errors, and then them talking about what actions to take from then on, which showed me how to overcome mistakes, just something to emulate.’ (FCSD, P2–99)*

When completing the FCSD, they began to perceive themselves actively seeking learning strategies used by model doctors or mentors, and adapted them to their self-study. They no longer had to rely on the teacher’s yardstick like test scores or pass/fail test results. Aside from a simple memorizing strategy, they began to apply a variety of learning strategies for what they perceived in their patient care or how admirable mentors and medical doctors prepared for patient care.

*‘As if doing actual treatment, in my case I kind of think how I could do it, looking at results from clinical questions and checking the literature, which is different from until I was a six year.’ (FCSD, P3–51)*

*‘I’m writing down summaries of all patients’ info on my own, and then making my own plans for the basic treatment for them (in my mind). I’m glad my plans are the same as the professors actually did, and seeking feedback by myself if I’m wrong.’ (FCSD, P-3-19-1)*.

These changes perceived by FCSD participants were made clear when we referred to perceptions of non-FCSD participants toward Q3. The non-FCSD participants perceived limitations with learning strategies like rote memorization while they continuously stayed in the teacher-centered curriculum.

*‘It’s not like I'm such a bookworm, but in the end, success meant becoming like the textbook.' (Non-FCSD, N2–32)*

The non-FCSDs needed to rely on the ‘absolute’ indicator of test scores or correctness of answers corresponding to teachers’ instruction.

*‘The only way I could figure out if I was learning anything was from exam and practice exam results, then going over material that I got wrong.' (Non-FCSD, N2–28)*

All in all, the FCSDs perceptions indicate contextual shift from a teacher-centered to a learner-centered program might improve self-reflection without too much dependence on test scores and increase diversity of learning strategies.

### How does SRL develop during transition from a teacher-centered to a learner-centered context?

We further explored by focusing more on contrast of three elements in SRL between those who experienced the transition and those who did not. We thoroughly reviewed codes from Q1 to Q3, and eventually we identified coherent and meaningful patterns in codes based contrasts between FCSDs and non-FCSDs. Codes were converted into three themes: 1) a motivational contrast between “as an individual with a future self-image” and “as one among a crowd set by the teacher’s yardstick”; 2) reflection on “between current and future selves” or “between selves and the teacher’s yardstick”, and 3) diverse or monotonous/homogeneous learning strategies.

#### Theme 1. Motivational contrast between “as an individual with a future self-image” and “as one among a crowd set by the teacher’s yardstick”

Overall, the most prominent feature of the FCSDs was an enriched description of ‘selves’ from the past to present and future, as an individual learner. The FCSDs described relevance between their past and present learning activities and their future professional roles.

*‘I just imagined myself going around in a group, just one among many, but then I began to take-off as an individual...the biggest change was that I began thinking that how far I want to go was really up to me, so then I could go and make the choices for my future.' (FCSD, P2–44)*

Their self-motivation reached a climax when FCSDs perceived themselves being treated as a responsible person on the same level in learning by mentors and surrounding professionals in medical practice.

*‘In the Free Course it was like I was given a lot of responsibility by the teachers which really motivated me.’ (FCSD, P1–38)*

On the other hand, the non-FCSDs were stuck in their performance within the values set by the teacher’s yardstick (e.g. assessment test scores, and pass/fail standards) and described themselves as ‘someone”, resulting in the scarcity of future self-image as a doctor.

*‘It was more like I was someone on a mission, rather than, you know, wow, I wonder what it would be like to work as a doctor.' (Non-FCSD, N1–36)*

They stated fear of failing tests strongly motivated them to undertake self-study. However, fear-based motivation only prompted them to seek the ‘safety zone’, where they could perceive themselves not left behind other classmates in a crowd set by the teacher’s yardstick (pass/fail threshold).

*‘It’s a safety zone. Since there’s no getting out of taking exams, I really only focused on placing in the “non-fail” range, not on getting a high score.’ (Non-FCSD, N1–39)*

#### Theme 2. Reflection on “between current and future selves” or “between selves and the teacher’s yardstick”

In the FCSD course, liberation from the absolute indicator set by the teacher’s yardstick eventually helped them identify an alternative indicator: distance between their current ability and achievable self-image. The FCSDs recalled a possible alternative indicator during self-study in the FCSD context. They attempted to set ‘their own indicator’ within themselves, for example, by measuring the smoothness of their medical practice in a self-reflective manner.

*‘From the outset, going from the first-time patient interview to the assessment...I was able to get the hang of it compared to before, and at the same time I kept reviewing how smoothly I interviewed her or how I was nervous and skipped some steps. (FCSD, P2–42)*

On the other hand, non-FCSDs also had reflective-like behaviors in their self-study but they did not perceive they needed to carefully evaluate their learning outcomes in a self-reflective manner or attempted to establish their own concrete indicators for their achievements. They seemed to blindly rely on referring to test score or pass/fail results determined by teachers.

*‘Well, what I usually did for better or for worse was kind of rely on my gut feelings, or else, you know, like test scores.’ (non-FCSD, N2–34)*

#### Theme 3. Diverse or monotonous/homogeneous learning strategies

In the teacher-centered context, undergraduates associated effort management for memorizing knowledge prepared by teachers with test success or at least survival. They studied using effort management on repeated memorization of textbooks or handouts from teachers, and sometimes they were demotivated by overwhelming memory workload.

*‘There were questions about surgery...but ultimately there was a lot of material I just didn’t get and couldn’t prepare for, so the next tests are going to be hell...no matter how much I looked at my textbook things just didn't click...overall, I just couldn't jump-start my motivation so I ended-up just ignoring a whole lot.' (Non-FCSD, N2–14)*

After completing the FCSD, they perceived the diversification of their learning strategies while undertaking drill exercises using test items with clinical vignettes. Instead of merely reproducing the information written in textbooks, or lecture handouts, they came to associate clinical vignettes with what they encountered or what model physicians experienced in real clinical practice (elaboration in MSLQ). They mentioned that they were eventually able to deepen their understanding of the relevant structured knowledge (organization in MSLQ). While answering test items during self-study, they began to convert the negative feeling of mistakes into the acceptance as a next learning subject, that could be referred to as control of learning beliefs in MSLQ.

*‘Before it was like, I'd be figuring out problems (in test items), I know that, I don’t know that, but now I have a much clearer idea of how I'm getting things wrong, I can analyze it...So now making mistakes is not so much of a big thing. If it happens, it's like, ok, let’s just pay more attention next time.’ (FCSD, P2–67)*

## Discussion

**Fig. 1:**
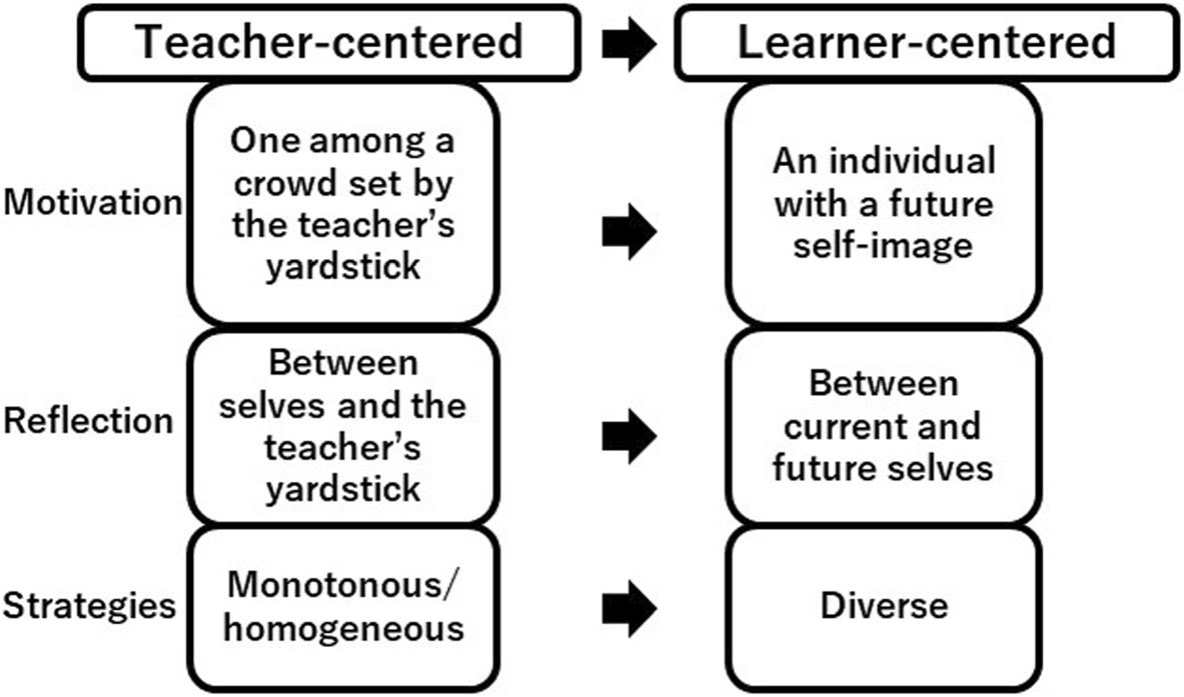
Changes in self-regulated learning from a teacher-centered (non-FCSDs) to a learner-centered (FCSDs) context in undergraduate medical education

To our knowledge, this is the first study specifically documenting contrast in SRL elements between undergraduates experiencing the contextual change from a teacher-centered to a learner-centered learning and those continuously remaining in a teacher-centered context. By incorporating the results of qualitative analysis for the two research questions, we concluded that learner-centered contexts could promote 1) motivational shift from “one among a crowd set by the teacher’s yardstick” to “an individual with a future self-image”; 2) reflection comparison from “between selves and the teacher’s yardstick” to “between current and future selves”; and 3) strategies from monotonous/homogeneous (memorization) to diverse (elaboration, organization, control of learning beliefs etc.) (Fig. 1). We found the possible link between formation of individual identity as an independent learner and eventual development of self-reflection and diverse learning strategies. Some theories may explain the linkage of identity formation and motivation-driven self-reflection and strategic learnings.

First, we employed the “constructive developmental theory” proposed by Kegan [32], which describes the process of identity formation and subsequent behavioral changes. According to Kegan, identity formation is composed of five stages, of which the second to fourth stages are particularly relevant to the learning period from undergraduate to postgraduate study in medicine [33].

At Stage 2, medical students still lack a broader understanding of what it means to be a physician, and their motivation and performance is based on a narrowly defined and superficial understanding. As they move to Stage 3, they begin to internalize social expectations, behaviors, and values of the profession, and become sensitive to whether they are doing things right as a physician. This emerging identity motivates them to learn rules of appropriate action and to look to authority figures for direction and reassurance that they are doing well and fitting in. In Stage 4, individuals construct a personal system of values and internal processes they use to evaluate external messages about their role and competence. Through this evaluation, they acquire the ability to think about themselves in relation to the larger system involving all medical professionals. The transition from stage to stage is not gradual but rather precipitated by emerging “identity crises” [34]. Namely, when faced with discrepancies between their understanding of themselves in the role and their understanding of experiences and challenges they are facing, they begin to reevaluate their situation, incorporate new information, and eventually develop a new understanding of the world or themselves [33].

In our present study, all the FCSDs articulated discomfort and anxiety of being exempt from teacher-centered values. On entering the FCSD context, they were accustomed to pursuing common learning goals set by the teacher’s yardstick, assuring they did not differ from classmates in order not to fail. However, the FCSD context pushed them to face discrepancies between the role of “as one among a crowd set by the teacher’s yardstick” and the challenge of having no prepared goals or assurance of their improvement in self-study. They recognized the necessity of finding alternative indicators within reach of their perception to assure themselves they were doing things right without making comparisons “between selves and the teacher’s yardstick.” Accordingly, they began to reflectively compare “between current and future selves.” In other words, such a crisis prompted them to ask themselves who they would like to be as an individual professional. While overcoming the discrepancy, they were likely to internalize how authority figures (mentors and role-models) behave by incorporating new learning strategies. This could result in diversification of learning strategies. Cruess et al. [35] emphasized importance of individual identity formation in medical education by referring to ‘professional identity formation (PIF)’, defined as formation of “a representation of self, achieved in stages over time during which the characteristics, values, and norms of the medical profession are internalized.” PIF results in an individual thinking and acting over what they want to learn and what they find important in a clinical environment [35]. All in all, these notions support contextual change toward learner-centered learning caused motivation to be based on the idea of an individual with a future self-image, and reflective comparison to be oriented to current and future selves. Accordingly, learning strategies were no longer limited by the teacher’s yardstick, and became diverse.

Second, the “self-determination theory” proposed by Ryan and Deci [36] could be employed to explain how the FCSD context promoted a shift in regulation of learning from controlled to autonomous. This theory states the degree an individual’s behavior is self-motivated depends on fulfillment of intrinsic needs for competence, autonomy, and psychological relatedness. In our present study, the FCSDs perceived the most advantageous aspect of their approach was the ability to decide one’s own learning plan and the opportunity to choose a tutor they admired and an institution where respected physicians work. Moreover, their self-motivation reached a climax when students perceived themselves as being treated by mentors and surrounding professionals as similarly responsible in learning. These features fulfill the need for autonomy (self-determination in learning activities), competence (being treated as a responsible person), and relatedness (close interaction between admired tutors and learners), and eventually made students more self-motivated.

In practice, the idea that contextual change from a teacher-centered to a learner-centered (individualized) learning positively influences SRL could be used as follows. We propose the undergraduate curriculum be designed in such a way that students more closely participate in planning of their content by self-determination with higher responsibility. The higher responsibility entailed by self-determination for their own learning might encourage them to think of their own learning activities more carefully and profoundly. Instead of having their learning outcomes all designed by teachers, they could develop their learning outcomes based on their reflection of how they would like to be in the future, and how they have missed learning in the past. From these points of views, the FCSD at JMU and self-proposed student-selected components in the UK [37] might be a good platform to give opportunities fostering PIF and SRL.

Of course, the undergraduate curriculum should certify the mastery of certain knowledge and skills. Because medical students are inaccurate in self-judgement of their knowledge, skills and performance [2], feedback is inevitable. One study found that individualized and narrative descriptive feedback from mentors promotes PIF elements [38]. Therefore, to optimize self-determination-oriented elective courses, we need to establish mentorship systems to provide individualized and narrative descriptive feedback on a regular basis. To maximize the effect of feedback, the ability of students as well as mentors to communicate with each other should be fostered sufficiently.

### Limitations and further research

A limitation of this study is its analytic comparison between two groups, which were each sufficiently competitive to pass the national licensing exam at the end of the second-to-last school year. However, the findings in this study would justify further investigation to explore whether a curriculum reform toward learner-centered learning could stimulate SRL in low-grade undergraduates, especially in teacher-centered education culture.

A second limitation is this study only investigated the retrospective notion of learning activities. We admit the possible uncertainty of qualitative data collected from participants’ recollection. However, both cohorts were composed of participants with higher grades than the average in the Year 5 recollection-dominated tests, and we only included those participating in the FCSD or the counterpart in the conventional curriculum within the latest 2 years in order to maximize the accuracy of recollection. Moreover, the contrast in SRL changes between those experiencing the contextual change and remaining in the teacher-centered curriculum ensures this contextual change could promote significant changes in SRL over the 7 months.

A third limitation is we did not directly evaluate SRL levels when they started the FCSD or decided to stay in the teacher-centered curriculum. Even though changes of SRL were identified between *before* and *during* the FCSD according to the focus groups statements for Q3, the present study design might leave the assumption FCSDs chose this student-selected elective course because they were self-motivated to enter new challenging environments to develop as individual learners.

Judging from the second and third limitation, a more valid approach to the research question can be to compare the SRL levels of the same individuals among pre-, peri-, and post-FCSD. Further investigation should be conducted in such a longitudinal manner.

A fourth limitation is that this study only focused on self-study for knowledge acquisition while a variety of learning activities take place in undergraduate settings. Self-regulation in learning is applied not only to self-study but also to learning in groups. Recent theories suggest that self-regulation in learning can be developed through social transactions, considered the central core of regulated learning [3, 4, 8]. In the context of our present study, for instance, undergraduates might develop SRL in a peer-group study rather than by self-study. Accordingly, future studies should focus on changes in regulation for learning through social interactions among participants in various learning settings.

## Conclusions

Allowing for these limitations and the need for further research, this study indicates contextual change toward learner-centered learning could promote SRL even in students strongly accustomed to teacher-centered learning. In the learner-centered context, students began to construct their future self-image, conduct reflection on current and future selves, and seek diverse learning strategies by referring to future ‘self’ models.

## Additional file

Additional file 1: Representative codes and statements. Representative codes and statements written in Japanese and English (PDF 769 kb)

## Abbreviations

FCSD: Free Course Student Doctor
MSLQ: Motivated Strategies for Learning Questionnaire
PBL: Problem-based learning
PIF: Professional identity formation
SRL: Self-regulated learning
