# Supplementary material for: Does changing from a teacher-centered to a learner-centered context promote self-regulated learning: a qualitative study in a Japanese undergraduate setting
Source: BMC Med Educ. 2019 May 17;19:152. doi: 10.1186/s12909-019-1550-x (PMC6525356; doi:10.1186/s12909-019-1550-x)
Supplement: Supplementary file 1 — Representative codes and statements. Representative codes and statements written in Japanese and English (PDF 769 kb) [file 12909_2019_1550_MOESM1_ESM.pdf]

Question 1 *Could you recollect your experience of self-study (of medical knowledge) during the FCSD or the same period in the conventional (teacher-centered) curriculum?*

Focus group	Reference number	Statement in Japanese	Coding	English translation	Coding (English)
non-FCSD	N1-17	授業出てさえいれば卒試そのまま勉強できるって感じで良かった。	授業依存的学習	It felt good knowing that just attending classes would help me study for finals.	Lecture-dependent learning
	N1-20	卒試に関しては、もうほぼ総括講義の資料から出ているっていうところ。も実際にあって	授業依存的な試験志向学習	It's true that what was covered by finals was pretty much in the handouts from the comprehensive lectures.	Lecture-dependent test-oriented learning
	N2-2	卒業試験に向けて、それぞれの科目を試験に向けて勉強するのが精一杯な１、2学期だったのかなというふうに思います。	精一杯な試験志向勉強	I really wanted to give everything studying for course and graduations finals in the 1st and 2nd trimester.	Full effort for test-oriented learning
	N2-4	ある意味国家試験重視でより心にゆとりを持たせたいという勉強と、	テスト不安を減らすための試験志向学習	For me, it was like I could keep a pretty relaxed focus studying for the national exams.	Test-oriented learning to lessen test anxiety
			⋮		
FCSD	P2-1	今までテストで例えば一問一答みたいなのだったのが、どんどん学問の裾野が広がるみたいで、どうやったら今後は総診をやりながら自分の知識の幅を広げていけばいいのかなっていうふうに考え…	自分の知識を広げる学習課題	Until now it was like each question had a set answer, but as the subject expanded, it was like, from hereon in to do a general internal medicine how am I going to expand my own knowledge…	Learning subjects broadening your knowledge
	P2-11	自分が地域に行って診療所とかでやるようなことは見れなかったからあんまりイメージが…いろんな家庭医の先生の、全然診療スタイルが違って…いろんな出会いがあって面白かったです。	イメージが具現化した多様な将来モデル	I really didn't have much of an image of what I should do when going back to my home region to practice…doctors on their home visits have so many different styles …it was interesting meeting so many different people.	Embodied imagery with diverse future models
	P2-20	それぞれのペースでそれぞれの置かれた環境で興味を持っていることも違うし、どう成長していくかも違うんだから…	多彩な自己像の形成	It's hard to tell how to develop professionally since each location's got a different pace or a different environment, different interests…	Diverse self-image formation
			⋮		

Question 2 *During that period, how did you motivate yourself, what strategies did you apply to learning, and how did you assess your understanding?*

Focus group	Reference number	Statement in Japanese	Coding	English translation	Coding (English)
	N1-27	試験はやっぱりあんまり落ちたくないなって気持ちはあったので、そうですね、それは１つのモチベーションではありましたけど。	テスト不安に基づく動機づけ	I really did not want to fail the exams, so yeah, that was one motivation.	Fear (or test anxiety)-based motivation
	N1-36	そのときのミッションみたいな感じでこなしていたような感じで、将来の医師として働くときにどうかとかいうのあんまり考えてなかったと思います。	将来の自己像が見えないミッション集団の一人	It was more like I was someone on a mission, rather than, you know, wow, I wonder what it would be like to work as a doctor.	One among a mission-oriented crowd lacking future-self image
	N1-39	安全圏。試験とかもそうだと思うんですけど、そうですね、上のほうというか、試験落ちないくらいのに居たいっていう感覚でやってたかもしんないです。	不合格を避けるための安全圏と自分との比較	It's a safety zone. Since there's no getting out of taking exams, I really only focused on placing in the "non-fail" range, not on getting a high score.	Comparison between selves and ones within the safety zone in order not to fail tests
FCSD	N2-6	学習方法なんですけれども、レジュメをもらってそれを見直して、あとはなんでしょう。国家試験のその科の問題を練習したりとかですかね。あとはそれぐらいですかね。	単一的な学習方略	When studying, I always checked back with the course summaries, and what else…a lot of practice tests for the national exam. That's pretty much it.	Monotonous learning strategies

non-	N2-9	とりあえず教科書読んで問題を解くっていうのの繰り返しで。	単一的な学習方略	Well, I just solved the problem questions from the textbooks again and again.	Monotonous learning strategies
	N2-14	手術方式はどうかっていう問題とかあったんですけれども…結局最後まで苦手意識が残って、結局勉強できなくて今回のテストですごい苦しんで…教科書を見ても自分の持っている教科書では対応のしようもなくて勉強が全くできてなかったところなので…モチベーションどころか勉強しようっていう感じも起きなくてスルーしてしまったところなので。	努力要請できないことによる動機の喪失	There were questions about surgery …but ultimately there was a lot of material I just didn't get and couldn't prepare for, so the next tests are going to be hell…no matter how much I looked at my textbook things just didn't click…overall, I just couldn't jump-start my motivation so I ended-up just ignoring a whole lot.	Insufficient effort leading demotivation
			⋮		
FCSD	P1-38	フリーコースで行ったときは結構任せてくれるというか、先生も任せるみたいな雰囲気があったので自分のやる気にもなって。	任せられる扱いでの動機づけ	In the Free Course it was like I was given a lot of responsibility by the teachers which really motivated me.	Entrustment-led motivation
	P2-39	テストみたいに客観的に伸びたっていう指標とかはあんまりないと思うんですけど…最初の２週間に目標とかを先生と共有して、最後のほうとかに先生とかに到達度を診てもらったり…	省察の援助要請	While I don't think there's any way to objectively measure how much I've learned, the first couple of weeks I set goals with the professor, and near the end received recognition for achieving them.	Help seeking for reflection
	P2-42	最初から断続的に自分が一応、外来とかでファーストタッチしてアセスメントプランまで…繰り返すごとに前よりは考えられるようになったかなとか、前よりはスムーズに問診できたかなとか、逆にこれだろうってシャイ書に思いすぎてまだ自分はそこまでスナップ（snap diagnosis）できるほど知識も経験もないのに、自分が大変になって省略しちゃったなとか、そういうのを自分で振り返ってた気がします。	自己省察方略の確立	From the outset, going from the first-time patient interview to the assessment…I was able to get the hang of it compared to before, and at the same time I keep thinking there's so much more to learn since I can't even make a snap diagnosis.	Establishment of self-reflection strategies
	P2-44	五年生のころって班でまわるのでわりと何人かいるなかの一人っていうイメージだったんですけど、実際に一人で飛び込んで行って…どこまでやりたいとかも全部自分次第になってきて、そうすると自分で選んでいっているわけだし将来、実際ここに自分はなにしにきたのかとか、自分はここでなにを学んでどう将来に繋げたいのかっていうのを全部のことを考えるようになったのが一番変わって…	何人かいる中の一人から現在と将来の中の自分へ	Up until my fifth year I just imagined myself going around in a group, just one among many, but then I began to take-off as a individual…the biggest change was I began thinking that how far I want to go was really up to me, so then I could go and make the choices for my future, and what was really the reason I came here, and how to connect what I was learning with how I wanted my future to be.	Motivational states from one among a crowd to an individual with a future self-imange
	P2-62	情報を見てどうやってそれを解釈して、どうやって目の前の人に適用するのかなっていうのがすごい難しいなと思って、どうすればいいんだろうって先生を見てたんですけど…なるほど情報の使い方もいろいろあるんだなっていうのを覚えました。	モデルの方略への眼差し	How am I supposed to handle all this info around me, all the people too, it was really hard at first and just tried to follow the teacher's lead at first… then it was like, oh yeah, there's lots of ways to process all the info, that's what I learned.	Attention to models' strategies
			⋮		

Question 3 *Between before and during the period, did you experience any change in terms of how you motivated yourself and the strategies you applied to learning and to assessing your understanding?*

Focus group	Reference number	Statement in Japanese	Coding	English translation	Coding (English)
non-FCSD	N2-28	本番とか模試とかでちゃんと正解できるかとか、間違えたらそこを復習するっていうそれぐらいのアウトプットの方法しか自分は持ち合わせていないので	試験評価以外にはない省察方略	The only way I could figure out if I was learning anything was from exam and practice exam results, then going over material that I got wrong.	No other self-reflection strategies except for test-assessment
	N2-32	ペーパー至上主義じゃないですけど、教科書になってくると多くしてるもん勝ちというのはあったので	記憶学習	It's not like I'm such a bookworm, but in the end success meant becoming like the textbook.	Memorization
	N2-34	自己評価の方法も模試の結果だったりだとか、あとは自分の感覚みたいなのでよくわかんないですけど、こんくらいでいっかみたいな感じでやって	試験評価以外にはない省察方略	Well, what I usually did for better or for worse was kind of rely on my gut feelings, or else, you know, like test scores.	No other self-reflection strategies except for test-assessment
			⋮		
	P1-65	私はお手本となる先生を見つけようと思って、科を回るたびに、カルテでも素晴らしいカルテを書く方とか、一人ひとりの患者さんにどう向き合っているかっていうのすごいお手本にしたい先生は必ずいらっしゃるので。	モデルの方略への眼差し	I thought I'd find a doctor who could be a model for me, who knew how to write really good patient reports and was really good with them on a one-on-one basis, because I knew there just had to be one like that.	Attention to models' strategies
	P1-75	多数の中の一人よりは責任のプレッシャーもあり、より積極的に実習に取り組むのはあると思うんですけれども。やっぱり受け身の姿勢のときに聞いていたことと自分が積極的に行った学びの中で得たことっていうのは、データが同じものを学んでても印象に残る具合が違うのではないかと思いました。	学習の自己責任感	I get now that there's this responsibility not many other people have, and it makes me be more proactive in my learning. I've discovered there's a big difference between being on the receiving end and listening to things being taught to me, and going out on my own and trying to learn things. The material's the same, but the impression you get is completely different.	Self-responsibility in learning
	P2-67	試験問題とか解いてて、前はなんとなくあってた間違ってた、知ってた知らなかったみたいな感じだったのが、今は個々からここまでの情報は解釈できてた、でもこれを見落としたから診断を間違えたとか、問題の間違えたところを分析できるようになった…なった気がする。だから本当に間違えても変にへこまなくなった。いま自分はこれができなかったから、この問題ができなかったのであって、逆にこれをちゃんと気が付けるように次気を付けていれば間違えないとか…	学習信条のコントロール	Before it was like, I'd be figuring out problems, I know that, I don't know that, but now I have a much clearer idea of how I'm getting things wrong, I can analyze it…So now making mistakes is not so much of a big thing. If it happens, it's like, ok, let's just pay more attention next time.	Control of leaning beliefs

FCSD

P2-70	みんなバラバラなことをしてるから前まで見たいに分かりやすく自分の相対的な位置を知る指標がなくなったぶん、確かに自分を認めて、尺度とか人と比べてじゃなくて自分の満足いったかどうかっていう感じにはなったかな。	自他比較から自己成長の比較へ	Everyone’s doing their own thing, so you can’t see what’s in front of you, what the markers for progress are, and I was really able to get rid of that mindset, I stop worrying about scores and recognize what I was doing for its own sake.	From self-other comparison to comparison of their progress
P2-79	自分の達成度というか、ここはまだ自分に足りなかったことなんだなっていうのを見えたのかなって気がします。	メタ認知の確立	I think I got a sense of what my achievements are, and what I still need to learn.	Development of metacognition
P2-84	その人がなにを考えてどう学んだのか見たいのが分かったら、自分のそれをいいなって思ってコピーして次やれば、そういう風に自分も勉強の方法がゲットできるかもしれないっていう意味で気になっている気がします。	学習方略の模倣	Now it’s like if I can observe someone who knows how to study and how they pick-up new things, I can try to emulate them, and eventually it will come to me as well.	Emulation of learning strategies
P2-92	公衆衛生はいままですげえ味気なく思ってて辛いみたいな、覚えるの大変みたいに思ってたのが、それが終わって帰ってきて試験前だったのもあって勉強したら、これって確かにこのときだったりとか、ああいうとき大事だよなみたいな色が変わって見えるようになった。	精緻化による動機付け調整	Before I had a really difficult time with public health, I didn’t really think it was for me, but when I got back and began studying for exams, I could kind of pinpoint my experiences, oh this time was important, that time was important, which was a big change.	Motivation control by elaboration
P2-99	先生たちですらなんていうか失敗もあって、モヤモヤしてる姿とかも見てて、それをどうネクストステップはこうするよ、みたいのを言っているのをみると、そうやってそこは乗り越えていけばいいんだみたいな、振り返りかたみたいな。	モデルの方略への眼差し	I could also see the profs screwing up sometimes and getting anxious about their errors, and then them talking about what actions to take from then on, which showed me how to overcome mistakes, just something to emulate.	Attention to models' strategies
P2-101	患者さんをマネジメントしていこうっていうふうになると、あれ、これってどうしたらいいだろうって思うことがすごく多くて、逆に何もわからないんだなってことを認識して、何もわからないって思ったのが学び…	将来に関連高い学習課題の中でのメタ認知の誘発	There are still a lot of questions going around my mind concerning patient management, but now it’s like I know that I don’t know, so I gotta go and learn.	Metacognition stimulated in learning subjects relevant to their future profession
P3-19-1	メモ帳に簡単なカルテを付けて自分の基本的プランを全部の患者さんにやるようにしてて。それで方針が先生と一緒だったらやっぱうれしいし、間違ったら自分なりにフィードバックしてっていう感じで…	モデル教員と自分の方針比較しフィードバックする臨床での新たな自己学習方略の確立	I’m writing down summaries of all patients’ info on my own, and then making my own plans for the basic treatment for them. I’m glad my plans are the same as the professors actually did, and seeking feedback by myself if I’m wrong.	Establishment of a new self-study strategy with feedback by comparing between their plans and models' ones in clinical practice
P3-51	実際に診療について、自分だったらどうやるかなって考えるときにクリニカルクエスチョンが結構出てきて、それを文献をとおして解決するっていう手法が５年生までとは変わったところかなって思います	既存のカリキュラムにはなかった、変わった学習方略の確立	As if doing actual treatment, in my case I kind of think how I could do it, looking at results from clinical questions and checking the literature, which is different from until I was a six year	Establishment of a different study strategy that one did not have in the present curriculum

P3-77	やっぱりだから、評価軸ないからだと思うんですよ。分かりやすい。僕らは、飼いならされている…テストっていう分かりやすい、あれを至上にして生きてきた受験エリートたちなので、それから解放されてフリーになってるからすごい不安になるんですよ	集団内の評価軸からの解放	So overall, you know, there’s no yardstick to measure yourself. We’re all part of that group of elite test takers, so at the beginning when you’re liberated from that framework it’s really mind-boggling, confusing.	Liberation from an in-group assessment framework

⋮
